# Type III secretion system chaperones: a helping hand for secretion

**DOI:** 10.1099/mic.0.001753

**Published:** 2026-08-12

**Authors:** Kyra C. Roepke, Alexia J. Galsworthy, Adam Agbamu, Isabella C. Sheldon, Antoine Hocher, Camilla Godlee

**Affiliations:** 1 Department of Biochemistry, University of Cambridge, England, UK; 2 Department of Pathology, University of Cambridge, England, UK; 3 Department of Genetics, University of Cambridge, England, UK

**Keywords:** type III secretion system, chaperones, effectors

## Abstract

The type III secretion system (T3SS) is a virulence mechanism commonly used by Gram-negative bacterial pathogens to deliver virulence proteins, known as effectors, into infected cells. The T3SS secretes a range of different substrates: first the needle subunits, then the translocon pore components and finally a pathogen-specific range of effector proteins. Each of these classes of substrates interacts with a corresponding class of bacterial chaperones, which are required for their efficient secretion. The requirement for these chaperones has been attributed to multiple functions, including preventing premature substrate activity, maintaining substrate stability in the bacterial cytoplasm and mediating substrate targeting and secretion hierarchy. Here, we bring together what is known about the function of T3SS chaperones in a range of different bacterial pathogens. Through analysis of the conservation of chaperone sequence and structure, we discuss how these proteins interact with and support the secretion of diverse substrates. Finally, we evaluate the extent to which chaperones are universally required for effector secretion.

## Introduction

**Fig. 1.**
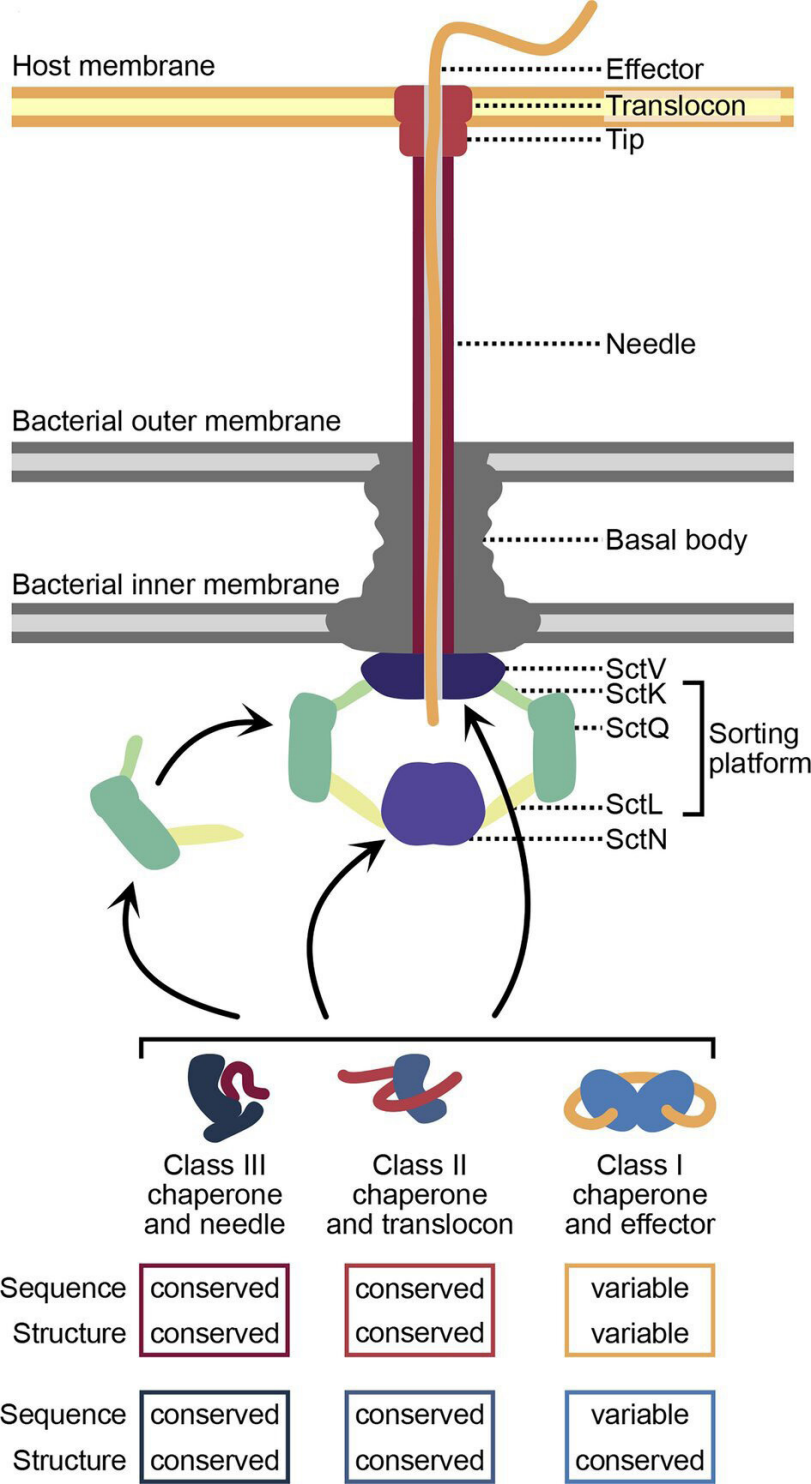
The T3SS and its chaperones. The major components of the T3SS injectisome. Needle, translocon and effector proteins are shown both in complex with their respective class of chaperone and within the secretion system. Cytoplasmic components of the secretion system suggested to be involved in chaperone:substrate recognition are indicated.

The type III secretion system (T3SS) is an important virulence mechanism used by many Gram-negative bacteria. It functions as a syringe-like injectisome to deliver effector proteins directly into host cells, where they manipulate host biology to enable bacterial survival and infection. The T3SS is evolutionarily related to the flagellum, and both consist of a basal body that spans the inner and outer membrane of the bacteria, enabling secretion of bacterial proteins. The T3SS secretes three classes of substrates: early, middle and late. Early substrates comprise the needle subunits that assemble into a hollow needle. The middle substrates are the translocon components, which form a pore in either the plasma membrane or an internal membrane of the eukaryotic host cell, and the hydrophilic tip protein that facilitates translocon assembly on the distal end of the hollow needle. Through this needle and translocon pore, a diverse set of effector proteins, the late substrates, are injected directly into the infected cell (Fig. 1). Each of these classes of substrates interact with a corresponding class of bacterial chaperones: class I chaperones interact with effectors, class II chaperones interact with the translocon components and class III chaperones are more broadly defined, interacting with external helical structural components including the needle and flagellin subunits. These chaperones associate with their clients in the bacterial cytoplasm and are required for their secretion but are not themselves secreted (Fig. 1, Table 1).

**Table 1.** Known chaperone:client pairs Class III only includes heterodimeric needle chaperones, with the large subunit listed first.

Class	Bacteria	Chaperone	Client(s)	Source
I	*Yersinia* spp.	SycE	YopE	[130]
	*Yersinia* spp.	SycT	YopT	[131]
	*Yersinia* spp.	SycP	YspT	[132]
	*Yersinia* spp.	SycO	YopO/YpkA	[45]
	*Yersinia* spp.	SycH	YopH, YsM1/LcrQ, YscM2	[133–136]
	*Shigella* spp.	IpgA	IcsB	[137, 138]
	*Shigella* spp.	IpgE	IpgD	[138, 139]
	*Shigella* spp.	Spa15	IpaA, IpgB1, IpgB2, OspC1, OspC2, OspC3, OspB, OspD1, OspD2	[123, 140–142]
	*Salmonella* spp.	SigE	SopB/SigD	[143]
	*Salmonella* spp.	SicP	SptP	[144]
	*Salmonella* spp.	SscB	SseF	[145]
	*Salmonella* spp.	SrcA	SteD	[41]
	*Salmonella* spp.	InvB	SipA, SopA, SopE, SopE2	[146–149]
	EPEC	CesF	EspF	[150]
	EPEC	CesT	Tir, Map, NleA, NleF, NleH, NleH2, EspH, EspZ, EspF, NleG, EspG	[151–156]
	*Pseudomonas aeruginosa*	SpcU	ExoU	[157]
	*Pseudomonas aeruginosa*	SpcS	ExoS, ExoT	[158, 159]
	*Pseudomonas syringae*	ShcA	HopPsyA	[160]
	*Pseudomonas syringae*	ShcM	HopPtoM	[161]
	*Pseudomonas syringae*	ShcV	HopPtoV	[162]
	*Pseudomonas syringae*	HrpG	HrpV	[163]
	*Chlamydia* spp.	CT584	CT082	[164]
	*Chlamydia* spp.	Mcsc	Cap1, CT618	[74, 165]
	*Chlamydia* spp.	Slc1	TARP, CT694, CT695, TepP, TmeA, TmeB	[164–166]
	*Xanthomonas* spp.	HpaB	AvrBs1, AvrBs3, XopR, XopS, XopB, XopE2, XopJ	[167–169]
	*Vibrio parahaemolyticus*	VecA	VepA	[12]
II	*Yersinia* spp.	SycD/LcrH	YopB, YopD	[24, 47]
	*Shigella* spp.	IpgC	IpaB, IpaC	[13, 21, 170]
	*Salmonella* spp. SPI-1	SicA	SipB, SipC	[42]
	*Salmonella* spp. SPI-2	SscA	SseC, SseD	[171]
	EPEC	CesD	EspD, EspB	[52, 172]
	*Pseudomonas aeruginosa*	PcrH	PopB, PopD	[173]
	*Chlamydia* spp.	Scc2, Scc3	CopB, CopD	[174]
	*Aeromonas hydrophila*	AcrH	AopB, AopD	[25]
Tip protein	*Yersinia* spp.	LcrG	LcrV	[29]
	*Salmonella* spp. SPI-2	SseA	SseB	[175]
	EPEC	CesA	EspA	[22]
	*Pseudomonas aeruginosa*	PcrG	PcrV	[176]
III	*Yersinia* spp.	YscG and YscE	YscF	[34]
	*Salmonella* spp. SPI-2	SsaH and SsaE	SsaG	[31]
	EPEC	EscG and EscE	EscF	[38]
	*Pseudomonas aeruginosa*	PscG and PscE	PscF	[37]
	*Chlamydia* spp.	CdsG and CdsE	CdsF	[177]
	*Aeromonas hydrophila*	AscG and AscE	AscF	[178]

The needle and translocon components are conserved across the different T3SSs of varied bacteria, as are their chaperones. Both chaperones and substrates are encoded alongside the core components of the secretion system, indicating their evolutionary conservation and importance. In contrast, effector proteins display a large amount of diversity in structure and function, both within individual secretion systems and across different bacterial species. The repertoire of effector proteins delivered by a species of bacteria defines its mechanism of infection. The genes encoding effector proteins are typically dispersed throughout the genome alongside their cognate chaperone, suggesting an evolutionary uncoupling between the secretion system and the effectors it secretes. Despite the extensive diversity and low sequence conservation of effector proteins, many class I chaperones share a conserved structure (Fig. 1). This raises the possibility that chaperone binding provides a common structural motif that enables otherwise unrelated effectors to be recognized by the T3SS. Yet paradoxically, many effectors lack an identified chaperone partner, and accumulating evidence suggests that chaperones are not universally required for effector secretion. In this review, we will discuss what is known about how chaperones recognize, stabilize and aid the secretion of T3SS substrates and discuss how universal the requirement for chaperones is for secretion.

### How do chaperones recognize their clients?

**Fig. 2.**
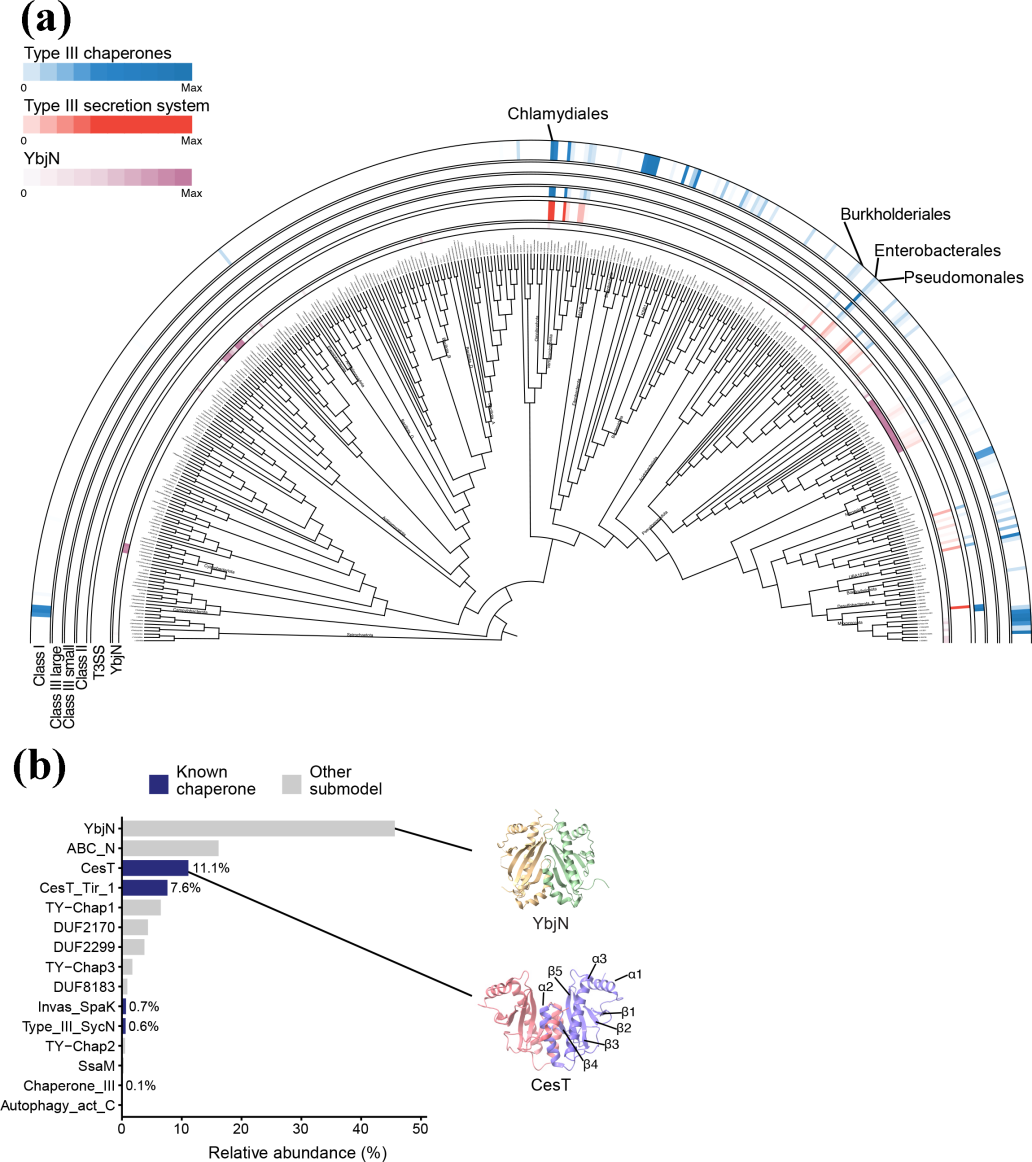
T3SS chaperone phylogeny. (a) Cladogram of bacteria, summarized at the order level as defined by the genome taxonomy database (GTDB) [179]. Outer heatmap rings indicate the frequency (%) of each protein family within each order in the database. T3SS presence was inferred using MacSyFinder. Class I chaperones were defined as the aggregate of functionally annotated chaperone models from Pfam clan CL0097, TypeIII_Chap: PF03519 (Invas_SpaK), PF05932 (CesT), PF07824 (Chaperone_III), PF21665 (Type_III_SycN) and PF22550 (CesT_Tir_1). YbjN, class III large, class III small and class II were defined using PF10722, TIGR02508, TIGR02501 and TIGR02552, respectively. (b) Relative frequency of each HMM from Pfam clan CL0097, TypeIII_Chap, computed across a database of ~65,000 phylogenetically balanced archaeal and bacterial genomes. Sub-families annotated with T3SS chaperone functions are coloured blue. Structures of YbjN (PDB 6VU7) and CesT (PDB 5Z38) are shown with each monomer in a different colour. Secondary structure elements are labelled for CesT.

To gain a broad view of the evolutionary relationships and conservation of the three classes of T3SS chaperones, we mapped the presence of the T3SS and their chaperones across bacteria (Fig. 2a). In line with the literature, we found that class II and class III chaperones are well conserved and have a phylogenetic distribution that aligns with that of the T3SS. Extensive homology within this class of chaperones allows insights gained from experimental data on one secretion system to be applied to others. In contrast, class I chaperones have a much more complicated phylogenetic history with extensive sequence variation such that clear structural homologues can have very limited sequence homology. This complicates their classification and hinders our understanding of their evolutionary relatedness.

### Class I chaperones

The low sequence conservation and complex phylogenetic history of class I chaperones raise the question of what features favour the repeated use of this structure by effectors. It is equally important to understand how this conserved structure can interact with such a wide range of diverse effectors.

Effector chaperones are split into two subclasses: class IA chaperones interact with a single effector and are often encoded next to this effector’s gene in the genome, while class IB chaperones are multi-cargo chaperones capable of binding several distinct effectors and are often but not always encoded alongside genes for components of the secretion system [1]. The line between these two classes is blurry, and chaperones once defined as class IA, such as enteropathogenic *Escherichia coli* (EPEC) CesT, have been reclassified as class IB upon discovery of additional effector binding partners. Interestingly, CesT has also been shown to interact with bacterial proteins that are not effectors, suggesting additional roles for these chaperones.

Many crystal structures of class I chaperone dimers have been solved, including CesT from EPEC and both SrcA and SicP from *Salmonella*. Class I chaperones are small (15–20 kDa) and acidic (pI 4–5). They form homodimers, with each monomer consisting of a small domain and a large domain [2–4]. The small domain is composed of an alpha helix (*α*1) and an extended loop region, which allows the helix *α*1 to fold to a different position relative to the larger domain in each of the two monomers. The large domain typically contains several twisted antiparallel beta sheets (*β*1-*β*2-*β*3-*β*5-*β*4) flanked on either side by alpha helices (*α*2 and *α*3). The hydrophobic surfaces of these alpha helices interact to form the dimer interface (Fig. 2b) [2].

Most class I chaperones are part of the Pfam clan CL0097. Within this broad protein family, members of at least five different Pfam sub-families are functionally defined as class I chaperones, while the rest of the clan is functionally divergent (Fig. 2b). Some known class I chaperones do not fall into any of these sub-families, and interestingly, some proteins that are part of the five chaperone sub-families are found in bacteria that do not contain a T3SS, suggesting that the sequence annotation might not clearly delineate the corresponding functional space. Proteins in the broader protein clan maintain a conserved 3D structure (Fig. 2b). Although predominantly prokaryotic, the clan also contains the PF03987 domain, which is found in eukaryotic proteins with functions in autophagy, such as the E2-like enzyme ATG10 in humans, potentially indicating horizontal gene transfer. Interestingly, the sub-family most represented across bacteria, YbjN, is surprisingly understudied and has only elusive links to chaperone activity (Fig. 2a). YbjN was initially characterized in *E. coli* as a stress response protein, as overexpression of YbjN helps suppress the temperature sensitivity of other proteins [5]. Two models have been proposed: (1) YbjN could slow cell growth to give the cells more time to unfold the heat-destabilized proteins and (2) YbjN itself may stabilize the unstable proteins. In *Deinococcus radiodurans*, the YbjN-like protein DR1245 has been proposed to function in DNA repair [6]. Further blurring the lines between sequence-based annotation and function, we note that a known *Chlamydia* class I chaperone, MscC (CT_260), is annotated as YbjN. It, therefore, seems that this 3D architecture might have been co-opted various independent times, suggesting a functional plasticity for this protein fold. High sequence variability could allow this protein family to be involved in varied protein interactions. Further work is required to better understand the evolutionary relationship of these sub-families and determine whether class I chaperone activity has evolved once or been obtained multiple separate times.

**Fig. 3.**
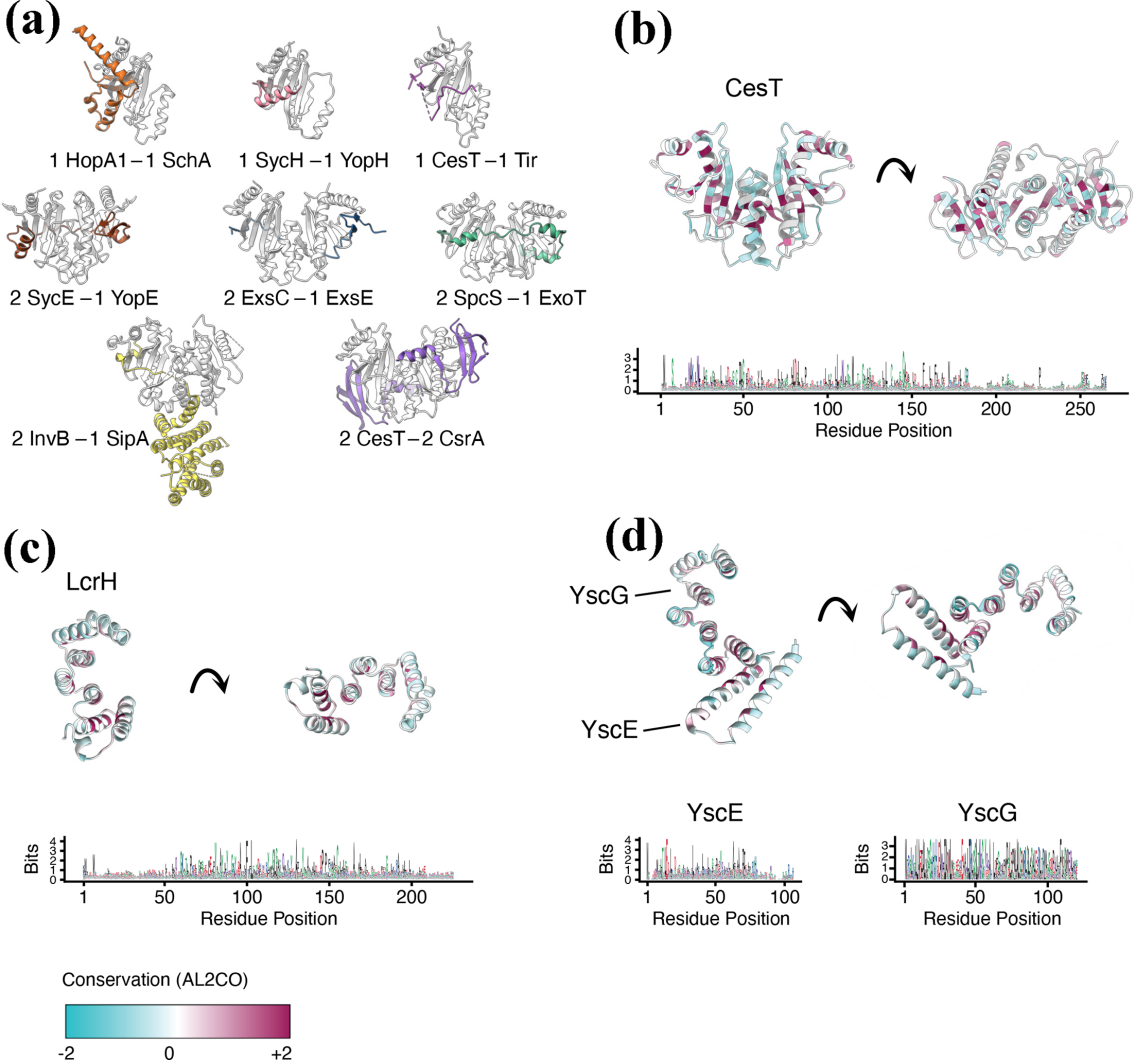
T3SS chaperone structures and conservation. (a) Solved structures of T3SS class I chaperone:client complexes (HopA1:SchA PDB 4G6T, SycH:YopH PDB 4GF3, CesT:Tir PDB 5WEZ, SycE:YopE PDB 1L2W, EsxC:ExsE PDB 3KXY, SpcS:ExoT PDB 4JMF, InvB:SipA PDB 2FM8, CesT:CsrA PDB 5Z38). Structures were aligned and visualized using ChimeraX. (b–d) Sequence conservation, visualized using ggseqlogo, of (b) class I, (c) class II and (d) class III T3SS chaperones, with relative sequence entropy mapped onto the corresponding experimentally determined structures (CesT PDB 5Z38, LcrH PDB 2VGX, YscE/G PDB 2P58). For each family, sequences were retrieved from our local database based on matches to published HMM models. Sequences were aligned using MAFFT and structures were visualized using ChimeraX.

Extensive work has been done over the years to characterize how T3SS chaperones bind to effectors. The structure of the class I chaperone dimer remains unchanged upon binding to its client [7]. Limited proteolysis of *Salmonella* chaperone SicP with its effector SptP and *Yersinia* chaperone SycE with its effector YopE has shown a stable N-terminal region on the client protein that is protected from degradation (residues 35–139 and 17–85, respectively) [8, 9]. This region, known as the chaperone-binding domain (CBD), defines effector:chaperone interactions. The CBD binds to the surface of the chaperone dimer, often wrapping around the outside of the complex. For SycE:YopE (PDB 1L2W), ExsC:ExsE (PDB 3KXY), SpcS:ExoT (PDB 4JMF) and others, the CBD forms a beta sheet extension from *β*1 at the distal end of the chaperone, far from the dimer interface (Fig. 3a). However, there is some diversity in the position of the CBD interaction with the chaperone dimer. Class I chaperones typically bind to their clients with a 2:1 stoichiometry, which means that the effector has different interactions with each of the two monomers in the chaperone homodimer. Other stoichiometries have been observed. For example, in addition to binding effectors, the EPEC class IB chaperone CesT also binds an RNA-binding post-transcriptional regulator, CsrA. CsrA forms a symmetric homodimer in its active state with two chaperone-binding sites. Therefore, *in vitro* complex formation of CesT:CsrA leads to irregular protein aggregations. In order to avoid oligomerization, a non-symmetric CsrA construct was designed for structural studies of a 2:2 CesT:CsrA complex [10]. As it is challenging to crystallize full-length effectors in complex with their chaperones, many structures show only the CBD of the effector interacting with a single monomer of the chaperone, forming a 1:1 complex in the crystal structure. The structure of the *Salmonella* SicP:SptP complex with a longer fragment of SptP shows a 4:2 binding structure in which each SptP protein wraps around two distinct chaperone dimers, with the two SptP proteins forming the classical beta sheet extension on opposite chaperone dimers from each other [8]. However, it is unclear whether this alternate stoichiometry is a crystallographic artefact. By contrast, for the EPEC pair CesT:CsrA (PDB 5Z38), the effector binding site is rotated and is perpendicular to the chaperone dimer interface, lacking the beta sheet extension that has been observed in many other structures. Interestingly, CBDs do not share sequence similarities, and the sequence of the chaperone:effector interaction interface is not well conserved, highlighting that the basis for their recognition by class I chaperones is still not fully understood.

A comparison of the sequences of class I chaperones in the CesT sub-family reveals that most of the sequence variation is at the surface of the chaperone dimer (Fig. 3b). This is consistent with the positions of effector interactions and aligns with the fact that the class I chaperone family interacts with a large range of effectors. In contrast, the most conserved parts of the dimer are internal within the chaperone dimer, especially in the *α*2 helix and *β*3-*β*4 strands at the dimer interface. These conserved sites are likely required for the quaternary structure of the chaperone dimer.

The CBD is the most important region for chaperone binding. For example, although the *Salmonella* effector SptP from *Salmonella typhi* shares 94% sequence conservation with that from *Salmonella typhimurium*, the majority of these changes are concentrated in the CBD. As such, SptP from *S. typhi* cannot interact with the *S. typhimurium* chaperone, but exchanging the CBDs between the two SptP proteins recovers this interaction despite the remaining sequence differences outside of the CBD [11]. Therefore, even small changes to the CBD can significantly impair chaperone:effector interactions within different serovars of the same species, while changes outside of the CBD are much less impactful. Despite this, the lack of homology in the sequence of the CBD makes it hard to predict how the interaction with the chaperone dimer is mediated.

Other than the CBD, much less is known about the structures of effectors in complex with their bacterial chaperones or indeed whether effector domains outside of the CBD are involved in chaperone interactions at all. It is well established that effectors have an N-terminal secretion signal, typically the N-terminal 20–25 residues, which is distinct from the CBD. These secretion signals generally lack secondary structure, and, therefore, it is likely that they are not involved in chaperone recognition or binding but rather that chaperone interaction might make the secretion signal accessible.

While most chaperone:effector structures show that the effector CBD winds around the outside of the chaperone dimer, the *Vibrio parahaemolyticus* effector VepA retains a surprising amount of its folded secondary structure when bound to its chaperone VecA. Similar to other effectors, an N-terminal region of VepA is particularly critical for chaperone binding, suggesting that the CBD lies in this region. However, rather than wrapping around the outside of the VecA dimer, the remainder of VepA, including the region N-terminal to the CBD and the C-terminal half of the protein, adopts a highly folded conformation and sits adjacent to the VecA dimer [12]. This is particularly notable because the N-terminal region of effectors contains the secretion signal, and this signal must likely be unfolded for it to be recognized for secretion. The existence of this novel VecA:VepA-binding structure complicates this hypothesis and suggests that there may be multiple mechanisms for secretion recognition: either the client wraps around its chaperone and leaves the secretion signal unfolded and flexible or the secretion signal can be folded as long as it remains solvent-exposed and able to be recognized by the secretion machinery, which may be the case for VecA:VepA. This is an open area of research that needs more attention.

### Class II chaperones

Class II chaperones bind to the major and minor translocon components prior to their secretion and assembly on the tip of the needle complex. Translocon components are conserved across secretion systems (Fig. 2a), and therefore, the structures of class II chaperone interactions with their clients are much more uniform than class I chaperone interactions with the extremely diverse set of effectors.

Class II chaperones typically form an asymmetric homodimer, containing tetratricopeptide repeats (TPR) [13]. The TPR motif is a 34 amino acid sequence present in tandem arrays of 3–16 motifs, which form curved antiparallel alpha helices that pack together to facilitate protein–protein interactions [14]. In *Shigella*, the solved crystal structure for the class II chaperone IpgC shows an all-alpha helical structure with eight helices per molecule. The TPR repeats form a curved 3D structure with a concave ‘palm’.

In the absence of their clients, class II chaperones form dimers. Mutation of the dimerization interface of the class II chaperone SycD to block dimerization but preserve three-dimensional monomeric folding prevents secretion of its client translocon proteins, YopB and YopD [15]. However, the *Yersinia* class II chaperone LcrH forms only a weak dimer, with a K _D_ of around 15 µM, and the energy barrier to dissociate the dimer is low enough that partially dissociated complexes are likely present in physiological conditions [16]. Although it is still unclear what the biological purpose of this partial dissociation of the chaperone dimer is, several hypotheses have been proposed. It might be important for the chaperone to switch between partially dimerized and fully dimerized states to facilitate binding to the client translocon protein. Alternatively, it is possible that some class II chaperones can function as either dimers or monomers, and the ability to shift between the two states creates a natural equilibrium based on stability. Indeed, many class II chaperones primarily chaperone in a monomeric state. For example, a dimerization mutant of the *Pseudomonas aeruginosa* class II chaperone PcrH is still able to stabilize its clients PopB and PopD in the bacterial cell, as well as facilitate their export out of the cell. However, while wild-type and dimerization-deficient PcrH have comparable half-lives in the presence of clients PopB and PopD, the dimerization mutant is significantly less stable than wild-type in the absence of PopB and PopD [17]. Therefore, it is possible that dimerization is more important for the stability of the chaperones in the bacterial cell than for their function.

Work has been done to investigate the structures of class II chaperones with their translocon components in *Pseudomonas*, *Shigella*, *Yersinia*, *Aeromonas hydrophila* and EPEC [18–22]. Class II chaperones often bind their translocon in a 1:1 ratio. However, it has been observed that a 2:1 binding ratio is possible for *Shigella* class II chaperone IpgC and its translocon client IpaB [21].

Most crystal structures of chaperone:translocon complexes only include short fragments of major or minor translocon components, containing a consensus P/VXLXXP chaperone-binding motif known as the ‘molecular anchor’, found N-terminal to the transmembrane region [13, 18, 23, 24]. The molecular anchor fits into the concave TPR ‘palm’ of the chaperone. Sequence analysis across the entire class II chaperone family reveals that this concave ‘palm’ is more conserved than the convex outer edge of the chaperone (Fig. 3c). This is consistent with the importance of this region for client binding and with the evolutionary conservation of the chaperone-binding motif of the client itself. This contrasts with class I chaperones, in which the low sequence conservation of their effector-binding surface reflects the diversity of their clients. A single class II chaperone can bind either the major or minor translocon proteins, demonstrating the versatility of class II chaperone binding [18]. However, the molecular anchor is not found in all translocon components, including those from *Salmonella* pathogenicity island 2 (SPI-2), *E. coli* and *Chlamydia*.

As these structural studies did not include the rest of the translocon proteins, it is impossible to know if any other regions of the protein are involved in chaperone binding. A solved structure of a larger segment of the *A. hydrophila* major translocon AopB with its chaperone AcrH shows that the CBD of AopB binds in the concave ‘palm’ as expected, but the hydrophobic transmembrane domains of AopB are also involved in binding, interacting with the convex surface of the chaperone [25]. Based on this structure, class II chaperones may act to prevent aggregation of these hydrophobic regions. Similar binding conformations may exist across other class II chaperones and their translocon component clients.

### Tip protein chaperones

The translocon is joined to the needle by the tip complex, which caps the distal end of the needle and acts as a scaffold to aid in translocon assembly [26]. In EPEC, tip proteins assemble into coiled-coil motifs that form a long filament, whereas in other species including *Shigella* and *Yersinia*, the tip complex is a homopentameric ring. While some tip proteins employ chaperones for their secretion, this requirement is not universal and many tip proteins lack chaperones [27]. Tip protein chaperones represent a case in which the definitions of chaperone classes become ill-defined. Historically, tip protein chaperones have been included within class III, which was defined as the broadest category to include all chaperones of structural components, including the flagella [7]. However, tip proteins are middle substrates, and their chaperones are structurally distinct from both class II chaperones of translocon components and class III chaperones of needle complex components. For this reason, tip protein chaperones have sometimes been referred to as class IV chaperones [28].

EPEC EspA and its chaperone CesA bind in a 1:1 stoichiometry, forming a heterodimer [22]. CesA has an extended alpha helical structure, with a long N-terminal helix and two shorter helices connected by shorter linker loops. The outer face of CesA has primarily charged residues while the inner face is mostly nonpolar, creating a hydrophobic groove where EspA fits as a helical hairpin. Structural studies on the *Yersinia pestis* tip protein LcrV with its chaperone LcrG affirm that the chaperone is all-helical and that these helices are bundled together only loosely [29]. While the C-terminal region of LcrG binds tightly to LcrV, the N-terminal region of LcrG is also suggested to bind to LcrV, indicating that there may be multiple binding interfaces. More structural work should be done on tip protein:chaperone pairs to investigate the diversity of these complexes.

For tip proteins lacking bacterial chaperones, self-chaperoning may be used to maintain tip protein stability prior to secretion. The N-terminal four helix bundles of *Shigella* tip protein IpaD and *Burkholderia pseudomallei* tip protein BipD share structural homology with the EPEC chaperone CesA and the putative *Yersinia pestis* class III chaperone YscE [30]. N-terminal truncation of IpaD leads to oligomerization in solution, suggesting that the internal folding and binding of the N-terminal domain is involved in keeping the full-length protein secretion competent. However, it is unclear whether this represents true chaperoning activity or whether the N-terminal domain is simply necessary for the intramolecular stabilization of the protein.

### Class III chaperones

Class III chaperones bind needle complex subunits prior to their secretion and oligomerization between the bacterial and host membranes. Most class III chaperones form heterodimers, with two different proteins coming together to form the chaperone complex. The larger of these subunits is structurally very similar to class II chaperones, with TPR motifs forming a concave ‘palm’. The smaller subunit is not related to other T3SS chaperones and is structurally distinct (Fig. 3d). As such, this smaller subunit is sometimes referred to as class V [31]. Homologous class III chaperone pairs have been identified across bacterial species, including PscE/G in *P. aeruginosa*, YscE/G in *Yersinia pestis* and AscE/G in *A. hydrophila* [32]. This conservation of chaperone structures stems from the fact that class III chaperones likely evolved with the needle complex itself, reinforcing that the presence of two distinct chaperones may be functionally important for stabilizing needle complex components.

Although efforts have been made to understand the structures of these class III chaperones in complex with their needle complex client proteins, little has been reported about the structures of the chaperones in the absence of clients. Phan *et al*. solved the crystal structure of the smaller class III *Yersinia* subunit YscE and found that it forms a dimer in solution, with each monomer composed of two antiparallel alpha helices separated by a flexible loop. The dimer is held together by hydrophobic interactions between the two monomers [33]. However, since YscE primarily functions in complex with the larger subunit YscG, it is unclear whether this homodimeric structure is present in bacterial cells – and if so, it is unclear what the structure of YscG on its own might be.

Chaperone binding of needle components involves the C-terminal half of the client. The *Yersinia* needle protein YscF primarily binds to the concave ‘palm’ of the larger subunit YscG and makes very little contact with the smaller subunit YscE. YscE binds to the N-terminal TPR motif of YscG, and it has been proposed that its function may be more related to stabilizing YscG or recruiting the complex to the secretion apparatus than directly stabilizing YscF [34]. These same structural elements have been observed for PscEFG in *P. aeruginosa* [32].

Therefore, all three classes of clients interact with their chaperones via a distinct chaperone-binding domain. For class I and II clients, the CBD is N-terminal, with the secretion signal sequence often appearing directly before the CBD. Class III clients such as YscF have a C-terminal chaperone-binding region but still contain a similarly flexible, unstructured N-terminal tail that likely acts as a secretion signal [34]. Despite extensive effort, structures of chaperone:client complexes containing full-length clients are still lacking for all three classes. Without this information, it is hard to assess the mechanism by which these chaperones stabilize and enable secretion of their clients.

### Roles of bacterial T3SS chaperones

**Fig. 4.**
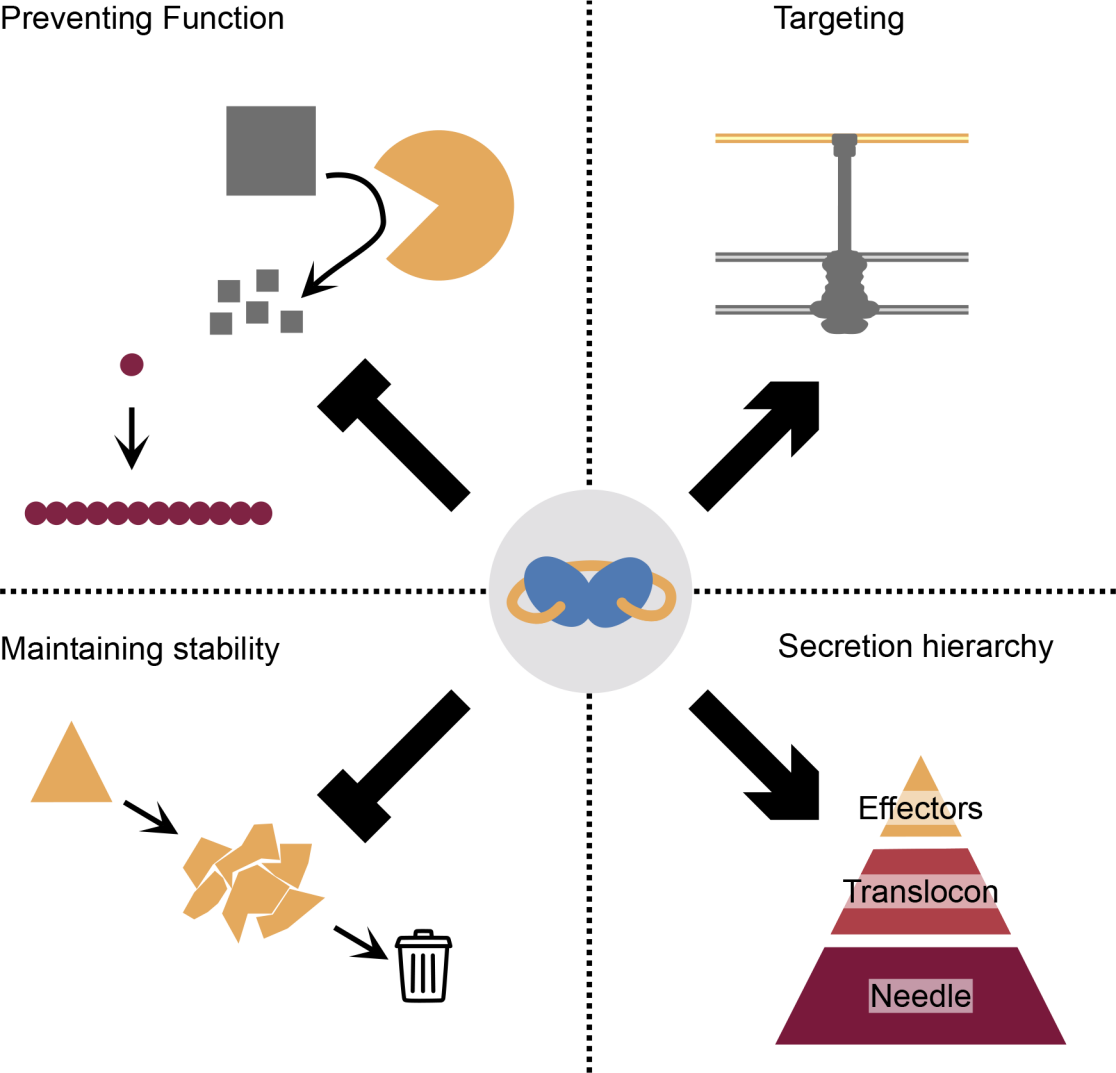
Models of possible roles of T3SS chaperones. T3SS chaperones have been suggested to be required for (1) preventing function by blocking enzymatic activity or self-assembly, (2) maintaining stability and preventing aggregation in the bacterial cell, (3) targeting substrates to the cytoplasmic side of the secretion system and (4) ensuring correct secretion hierarchy.

For many TS33 substrates, deletion of its chaperone prevents secretion of the client. This could be because the chaperone interaction is essential for its client to be recognized for secretion. However, there is also evidence that bacterial chaperones are important for blocking client function before secretion, maintaining client stability and regulating the hierarchy of client secretion (Fig. 4).

### Preventing function

T3SS substrates have specialized roles that enable bacterial pathogenesis. The needle and translocon components form the injectisome that connects the bacterial cell to the host cell, while effectors manipulate eukaryotic processes to enable infection. One possible role of T3SS chaperones is to prevent their client proteins from carrying out these functions before secretion.

Translocon, tip and needle complex components can readily self-assemble on their own [35]. Therefore, a major role of class II and class III chaperones is to prevent premature oligomerization of these proteins in the bacterial cell prior to secretion. The *Pseudomonas* chaperone PcrH and the *A. hydrophila* chaperone AcrH have been shown to block translocon assembly of their clients [25, 36]. Similarly, the main component of the *Pseudomonas* needle complex PscF readily oligomerizes on its own, but when bound to its two chaperones PscE and PscG, it is unable to assemble the needle structure [37]. The same has been observed for *Yersinia* YscEFG and EPEC EscEFG [34, 38]. Since chaperones are not secreted, the needle complex and translocon components can self-assemble following secretion.

For effectors whose role is to manipulate the host cell, chaperone binding could block the function of effectors while in the bacterial cell. Transmembrane effectors insert into host membranes following secretion. Deletion of the class I chaperone SscB in *Salmonella* causes its transmembrane effector client SseF to insert into the bacterial inner membrane, suggesting that chaperone binding plays a role in preventing transmembrane effectors from being recognized as transmembrane proteins in the bacterial cell. The *Salmonella* chaperone SscB was found to bind specifically to the first transmembrane segment of SseF, which may help prevent its recognition by bacterial membrane insertion pathways such as the SRP pathway [39].

Likewise, for effectors with enzymatic activity, chaperone binding may prevent the effector from having an active conformation in the bacterial cell. This is the case for *Salmonella* SptP, which functions as a tyrosine phosphatase in the host cell but lacks enzymatic activity in the bacterial cell [11]. However, this is not universally true for other effectors. The *Yersinia* effector YopE is catalytically active in *Yersinia* even when bound to its chaperone SycE, and in fact, the enzymatic activity of SycE:YopE is almost identical to the activity of unbound YopE despite SycE contributing no enzymatic activity [9]. The NleB class of effectors in EPEC and *Citrobacter rodentium* was shown to retain their catalytic activity in the bacterial cell [40]. However, as no chaperone has yet been identified for these effectors, it is unclear whether they retain their function during interaction with a bacterial chaperone or because they lack bacterial chaperones. Notably, for effectors whose only substrates are eukaryotic, preventing their enzymatic function in the bacterial cytoplasm might be unnecessary.

### Maintaining stability

Many T3SS substrates are not stable in the bacterial cell when their chaperone is deleted. Experiments studying substrate stability often involve overexpression, which could increase their aggregation propensity. Nevertheless, there is significant evidence that a possible role of T3SS chaperones is to prevent aggregation or subsequent degradation of their client prior to secretion. Transmembrane T3SS substrates, including transmembrane effectors and translocon components, have aggregation-prone hydrophobic domains. The *Salmonella* transmembrane effectors SteD and SseF and the *Salmonella* translocon proteins SipB and SipC have significantly shorter half-lives in the bacterial cell following deletion of their respective chaperones compared to in the presence of their chaperones [39, 41, 42]. Additionally, the *Yersinia* chaperone SycE prevents aggregation of the transmembrane effector YopE in solution [9]. However, since YopE secretion is still possible in the absence of SycE, it is possible that a certain amount of effector is still stable without the chaperone [43].

Even for the moderately hydrophobic membrane-associated *Yersinia* effector YopO, chaperone binding is essential for effector stability and solubility. YopO has a short N-terminal membrane localization domain and a soluble serine-threonine kinase domain [44]. Wildtype YopO is insoluble in the absence of chaperone but can be made soluble by either truncating off its hydrophobic CBD or by co-expression with its chaperone SycO [45]. Additional delineation between the CBD and the membrane localization domain shows almost exact overlap between the two regions, suggesting that SycO specifically protects the hydrophobic membrane-associated region of YopO to prevent aggregation in the bacterial cell.

Interestingly, there is evidence that chaperones also maintain the stability of fully soluble cytoplasmic T3SS substrates in the bacterial cell. For example, when the CBD of the soluble *Salmonella* effector SptP is disrupted to prevent binding to its cognate chaperone, it cannot be detected in the *Salmonella* cell [11]. However, not every T3SS substrate has a known bacterial chaperone. It is not yet understood what proportion of these substrates have as-of-yet undiscovered bacterial chaperones or whether they have a chaperone-independent mechanism for maintaining their stability prior to secretion. Thus, a question remains: what makes chaperone-dependent effectors different from those that are stable in the bacterial cell – and may even be successfully secreted – in the absence of any known T3SS chaperone?

### Facilitating secretion

Across a breadth of bacterial systems, chaperones of all three classes are shown to be necessary for the secretion of their T3SS substrates [41, 46–52]. The previously discussed role of chaperones in maintaining a stable population of many T3SS substrates in the bacterial cell could explain their requirement for substrate secretion, with the N-terminal secretion signal providing the information required for targeting to the injectisome. Alternatively, chaperones may be required to target their clients for secretion.

In support of a central role for chaperones in targeting, mutants of the *Shigella* effector IpgB1 that maintain their intact N-terminal secretion signal but lack their chaperone-binding domain are stably expressed in the bacterial cell but not secreted [53]. It is, however, possible that this mutation affects the conformation or accessibility of the secretion signal. As previously discussed, interaction with the chaperone could ensure that the N-terminal secretion signal is accessible and recognizable by the secretion system. Although secretion signals are often enriched in polar residues and depleted in charged or hydrophobic residues [39], effectors have a wide diversity of sequences and structures. Therefore, it is not clear how they are recognized for secretion. In contrast, chaperones do share conserved structural elements that could facilitate the recognition of this diverse range of effectors. It has, therefore, been hypothesized that the three-dimensional structure of the chaperone:effector complex forms a common structural motif that facilitates targeting to the T3SS injectisome [9, 54–56]. As such, is it possible to fully deconvolute the importance of the N-terminal secretion signal from that of the CBD? Phylogenetic analysis shows that proteins with a similar 3D structure to class I chaperones have a very broad range of functions, including functions outside of the context of secretion (Fig. 2a), suggesting that this 3D structure did not solely evolve for facilitating secretion and cannot be sufficient for secretion. This dichotomy then calls into question why effectors consistently require chaperones with this 3D structure for the secretion process.

### Targeting substrates to the secretion system

Secretion of T3SS substrates requires two carefully regulated and intertwined processes to occur: substrates must be physically targeted to the secretion system, and substrates must be temporally regulated to ensure that they are secreted in an appropriate order. The recognition of the chaperone:substrate complex by the secretion system, and the subsequent handoff of the substrate from the chaperone to the secretion system, are critical for secretion. This recognition may occur at different locations on the cytoplasmic side of the secretion system, including (1) the ATPase SctN; (2) the sorting platform, which consists of SctQ, SctK and SctL; or (3) the export gate SctV (Fig. 1). In this review, we use the secretion and cellular translocation (Sct) prefix to unify the names of these elements across all bacterial species, as reviewed by Deng *et al*. [57] and Wagner and Diepold [58].

The ATPase SctN has been implicated as a key component for chaperone:substrate recognition. It is hypothesized that chaperone:substrate complex interactions with SctN result in ATP hydrolysis, which provides the energy required to change the conformation of the chaperone:substrate complex. This enables the stripping of the substrate from the chaperone and allows for substrate secretion [59–62]. The SctNs from *Salmonella* SPI-1 and SPI-2 have been shown to dissociate chaperone:effector complexes *in vitro* in an ATPase-dependent manner [59, 63]. Proteomic approaches complement these biochemical studies [2, 64]. However, the structural elements of the chaperone:effector complex that facilitate this targeting are not well understood. Chen *et al*. dissect this problem and suggest that there is a substrate-activated conformational switch in the chaperone which increases its affinity for SctN. The authors show that the unbound EPEC tip chaperone CesA has a low affinity for SctN, but upon binding its client EspA, extensive refolding of CesA results in a CesA:EspA complex with high affinity for SctN [49]. Curiously, the class I EPEC chaperone CesT was found to be an ancillary factor for interactions between its substrate Tir and the ATPase SctN, suggesting that the chaperone instead aids in increasing the efficiency of substrate:SctN interactions [65].

Roblin *et al*. report a novel structure for the *Salmonella* chaperone:effector complex SopB:SigE, in which SopB binds to the effector forming a 2:1 stoichiometry typical of class I chaperone complexes, but as the concentration of chaperone:effector complex increases, the complex hexamerizes to form a ring. This ring structure is only found when the effector is bound to the chaperone and cannot be recreated in the absence of SigE. The authors hypothesize that it may help effector recognition by the ring structure of SctN at the base of the needle complex, perhaps by ensuring that the N-terminal tails of the effectors are all accessible [66]. This is the first evidence of chaperone complex oligomerization *in vitro*. Further research is needed to determine whether this oligomerization happens in physiological conditions in bacterial cells, as well as if similar oligomerization can be observed for other chaperone:client pairs.

There is also emerging evidence that chaperone:substrate complexes interact with elements of the sorting platform (SP). The SP is a cytoplasmic complex that plays a key role in substrate recognition and the recruitment of substrates from the bacterial cytosol to the secretion machinery. Cryo-electron tomography has revealed a distinctive cage-like architecture of the SP composed of six peripheral ‘shuttle’ pods of SctQ family proteins arranged around the central hexameric ATPase SctN [57, 67]. The SctO stalk protein anchors the ATPase to the export apparatus. The pods are connected to the ATPase via radial spokes of SctL and anchored to the inner membrane ring through the adaptor SctK, forming a dynamic interface between the cytosol and the export gate (Fig. 1). Importantly, the components of the SP, including the ATPase, are not permanently fixed to the basal body [55, 68–71]. These proteins also exist in the cytosol as soluble monomers or smaller subcomplexes and can dynamically transition between cytosolic and injectisome-associated states. Due to this intrinsic structural flexibility, knowledge of the structure of the SP lags behind that of the needle complex, which remains stable throughout the harsh purification steps required for imaging via single-particle cryo-EM [72].

The dynamic exchange between cytosolic and injectisome-associated states has been suggested as a mechanism for targeting substrates to the T3SS. In *Salmonella*, translocon and effector T3SS substrates interact with SP components SctK, SctQ and SctL, while the removal of the chaperone-binding sites of these substrates abolishes their recruitment to the SP [55]. Similarly, studies in *Chlamydia*, *Xanthomonas* and *Yersinia* have revealed that the chaperone:substrate complex interacts with SctQ, resulting in interactions with SctN [70, 73, 74]. Whether chaperone:substrate complexes interact with these SP components directly to form tripartite chaperone:effector:pod complexes or instead in a substrate handover event without chaperone:pod interaction is a question currently at the frontier of this field. Recently, Pintor *et al*. investigated this question after discovering a direct interaction between SctQ, SctL and effectors in the cytosol of *Yersinia enterocolitica* [70]. By using *in situ* single-particle tracking photoactivated localization microscopy in live bacteria to measure diffusion rates of the class I chaperones SycH and SycE, their effector clients and SctQ, the authors were able to deduce that these chaperones do not form stable, specific interactions with the cytosolic sorting components. Therefore, they propose a new model in which chaperones transfer their clients to the SP.

Importantly, it has become clear in the past 15 years that the secretion system and SPs are more dynamic in nature than initially appreciated [55, 71, 75]. As such, it has been hypothesized that changes in dissociation affinities between SP elements and different chaperone:substrate complexes under differential environmental stress conditions may also facilitate an additional regulatory layer to T3SS secretion [71, 76–79].

Finally, the chaperone:substrate complex has been shown to interact directly with the export gate SctV, forming chaperone:substrate:injectisome ternary complexes. While evidence for this is more limited, Gilzer *et al*. provide structural evidence for an interaction in *Yersinia* between SctV, the chaperone YscY and YscX (a middle substrate in *Yersinia* with an unknown function) [80, 81]. The crystal structure shows that SctV forms a nonameric ring that interacts with the YscC:YscX heterodimer, with the N-terminal section of YscX occupying a binding site on SctV. Additional studies affirmed the importance of YscX’s N-terminal region for secretion [82]. The crystal structure shows that the C-terminal region of YscX inserts between protomers of the SctV ring, near the SctK binding site that couples the export gate to SctN. This model implies that SctV forms a docking system for the chaperone:effector complex, enabling chaperone handover via SctN’s ATPase activity, complementing existing SctV:SctN interaction models [77, 83, 84].

These putative mechanisms of chaperone-mediated substrate targeting to the T3SS for secretion are not mutually exclusive. For example, it would be possible for some chaperone:substrate complexes to directly interact with SctN, whereas different chaperone:substrate pairs may first interact with SctV. It is likely that in the majority of cases, handover occurs through ATPase activity by SctN to enable release of the substrates from the chaperone and into the injectisome.

### Secretion hierarchy

Components of the needle complex must be secreted first to assemble the needle, followed by the translocon components, which form the translocon pore into the host membrane through which effectors are translocated. T3SS substrates are therefore classed as early (needle), middle (translocon components) and late (effectors) substrates based on their temporal order of secretion. A range of factors affect the timing of T3SS substrate expression and secretion. These processes are primarily impacted by environmental triggers, such as cell contact, temperature and pH, as well as transcriptional regulation, post-transcriptional regulation and conformational changes to T3SS components that affect binding of substrates and their cognate chaperones [57, 85–88]. Two key proteins, SctU, an autoprotease, and SctW, a gatekeeper protein, regulate the switch between early and middle substrates and middle and late substrates, respectively. Here, we focus on the role of chaperone interactions in this complex regulatory system.

At the base of the basal body, SctU forms a component of the export apparatus. Initially, it was proposed that interactions with the needle length regulator SctP modulated SctU autocleavage, which activated a switch in secretion from early to middle substrates [88, 89]. However, autocleavage itself may not be the temporal trigger for substrate switching, as it has been shown that SctU autocleavage occurs after protein folding and before SctU is incorporated into the T3SS [90]. Thus, autocleavage may be necessary to confer the structural flexibility required for SctU function rather than a molecular switch.

Relatively little is known about how needle complex components and their class III chaperones facilitate interactions with the export gate. However, it has been established that the smaller subunit of class III complexes, which might recruit the complex to the secretion system, can also interact with class II substrates. For example, the *Salmonella* smaller subunit SsaE forms a stabilizing complex with both the larger class III subunit SsaH and the translocon component SseB [31]. Perhaps the structural homology between class II chaperones and the larger class III subunit allows for the smaller class III subunit to facilitate client handoff for both chaperones. More research is needed to understand how the export gate differentiates between early and middle substrates.

The switch from middle to late substrates is mediated by the gatekeeper protein SctW. It has been observed that mutations in SctW lead to impaired secretion of translocon components and hyper-secretion of effectors [86]. In *P. aeruginosa*, it has been suggested that SctW binds the ATPase SctN during early secretion, increasing its affinity for early substrates, then dissociates and binds the export apparatus, where it mediates the switch from middle to late substrates [89]. SctW activity has been studied using an innovative *in vitro* system with inverted inner membrane vesicles containing EPEC type III export apparatuses [91]. This work suggests that the binding of a complex including EPEC SctW to the export gate increases the affinity for translocon:chaperone complexes, promoting their secretion. In contrast, dissociation of SctW from the export apparatus led to high-affinity interactions with effectors.

Interestingly, chaperones play a key role in the temporal regulation of secretion by linking SctW to translocon components. In *Chlamydia*, SctW can form a complex with the class II chaperone Scc3. When Scc3 binds SctW, the translocon binding pocket of Scc3 is left unoccupied, facilitating formation of a gatekeeper:chaperone:translocon ternary complex [92]. This physical link could facilitate recruitment of translocon components to the injectisome directly, contributing to the temporal organization of secretion. Mutations in *Shigella* SctW that abolish binding to the class II chaperone IpgC disrupt secretion of translocon components and cause early and elevated secretion of effectors [92]. Additionally, in the absence of SctW, binding of translocon components to the SP is abrogated in *Salmonella*, despite unchanged cytoplasmic levels of the translocon components [55]. Thus, the interaction of SctW with class II chaperones appears to be a crucial element of secretion hierarchy.

Beyond gatekeeper activity, hierarchical secretion of translocon components prior to effectors can be linked to activity of the cytosolic SP. Lara Tejero *et al*. identified a direct interaction between the translocon components, effectors and SP components of *Salmonella*. In a ‘queuing’ mechanism, the SP only binds effectors in the absence of translocon components [55].

Regulation of the sequence of effector secretion is not the result of molecular switches, as is the case for injectisome assembly. Rather, it is the result of a combination of transcriptional timing, transcriptional regulation and chaperone:effector complex affinity for the ATPase, SP and export gate. Additionally, competition for free chaperone may drive the order of effector secretion in the case of multi-cargo class IB chaperones. In EPEC, the chaperone CesT is required for secretion of at least nine effector proteins [93]. The first effector to be secreted is Tir, which can bind CesT via both its N- and C-terminal regions, likely outcompeting other effector proteins for CesT binding and secretion [3]. After the effector pool has decreased and free cytosolic CesT has increased, CesT sequesters its non-effector client CsrA. As CsrA is a transcriptional repressor of another CesT effector, NleA, this interaction facilitates NleA expression [94]. Another layer of complexity is added by CesT post-translational modifications, which affect CsrA binding. Recently, it has been observed that two other T3SS effectors, NleH1 and NleH2, can act as serine phosphatases when in complex with CesT, which impairs its ability to bind CsrA [95]. It is not clear how chaperone-independent models of secretion fit into this chaperone-based hierarchy of effector secretion. However, it is possible that effectors without bacterial chaperones might be outcompeted by those that do have chaperones. Alternatively, the secretion hierarchy of effectors may to some extent reflect the expression and abundance of effectors in the bacteria as opposed to any active level of regulation [96].

### Maintaining secretion competence

Once the chaperone:client complexes have been successfully targeted to the T3SS machinery, the clients must be threaded through the needle complex. In order to fit through the narrow pore of the needle complex, which ranges in diameter from around 1.5 to 2.5 nm [27], secreted proteins must completely unfold and enter the needle complex as a linear chain of amino acids [97].

The process of substrate unwinding and translocation through the T3SS is an energy-dependent process. Alongside its role in chaperone release, the ATPase SctN is suggested to be the key factor in energizing substrate export [59, 98]. However, subsequent work has demonstrated that proton motive force (PMF) drives T3SS secretion [99–101], suggesting that while SctN acts as a target for chaperone:effector binding and facilitates chaperone release, PMF supplies the dominant energetic force for unfolding and translocation.

It is possible that chaperones aid in the process of substrate unfolding by keeping the client in a secretion-competent state. Upon binding its chaperone SycE, the *Yersinia* effector YopH changes from a globular to an extended conformation, wrapping around the chaperone, perhaps to facilitate threading through the needle complex [102]. Additionally, effectors have to be mechanically labile to be secreted. This would explain why tightly packed proteins such as GFP or ubiquitin (which have similar thermodynamic stabilities to effector proteins but are much more mechanically robust) cannot be secreted. The *Pseudomonas syringae* effector AvrPto is small and has a relatively unstructured cytoplasmic state, which likely contributes to its high mechanical lability, allowing it to be delivered through the needle complex without the assistance of a chaperone [103].

### Requirement for host chaperones post-secretion

Bacterial T3SS chaperones are not co-translocated with their clients [59, 104]. This raises the question of how these clients, which have been unfolded prior to translocation via the injectisome, can refold or be targeted in the host cytoplasm, where they have not co-evolved with targeting pathways or dedicated chaperoning systems. This question is especially significant considering that, as discussed, many T3SS substrates are not stable in the bacterial cell without their bacterial chaperones.

Translocon and needle components can readily self-assemble [35]; a key role of bacterial chaperones is to prevent this self-assembly in the bacterial cell prior to secretion. After being secreted, translocon components must insert into host membranes. These proteins contain hydrophobic domains, and while insertion into the membrane bilayer is thermodynamically favourable, the translocation of flanking hydrophilic domains across the membrane is not. This process may, therefore, be aided by the tip complex of the needle, which serves as a scaffold for the integration of the translocon components [105]. Other factors that promote integration include the acylation of translocon components [106, 107], and the presence of a C-terminal amphipathic helix of the major translocon that interacts with local negative charges from lipid head groups in the bilayer [25, 108].

How T3SS effectors are stabilized and successfully localize in host cells, however, is more diverse and generally less well characterized. While soluble proteins sometimes require chaperones to fold correctly [109], chaperoning is particularly important for transmembrane proteins, whose hydrophobic transmembrane domains are prone to aggregation prior to membrane integration. Typically, immunoprecipitation coupled with mass spectrometry [110–112] or yeast two-hybrid screens [113–115] have been used to discover novel host protein:effector interactions. However, no host proteins have yet been identified as chaperones for effectors by promoting their folding or stability. Interestingly, binding of phosphoinositides to the N-terminal phosphoinositide-binding domain of a group of effectors has been shown to promote their folding after delivery into host cells [116]. It may be possible that other host factors are hijacked to assume roles analogous to bacterial chaperones post-translocation.

There are some instances in which effectors have been shown to interact with host factors for their successful targeting in the host cell. For example, NleA/EspI, effectors of pathogenic *E. coli* and *Citrobacter*, localize to the Golgi apparatus via interaction with the COPII component Sec24 [117]. They stabilize Sec24 at the Golgi and, therefore, suppress intracellular trafficking, including the trafficking of major histocompatibility complex class II (MHCII) molecules to the surface of infected cells [118]. The *Salmonella* effector SteD hijacks adaptor protein complex-1 for its trafficking to endosomal compartments, particularly compartments containing MHCII molecules [119]. There, SteD facilitates the degradation of MHCII molecules, suppressing the host adaptive immune response [120]. Although both effectors localize to the Golgi, it is not clear how they are targeted there.

### Chaperone-independent models of secretion

Despite the apparent importance of bacterial chaperones for the secretion of T3SS effectors, there is clear evidence that questions the central role of chaperones in the stability and secretion of their clients. Although the secretion of YopE is greatly reduced in the absence of its chaperone SycE/YerA, it can still be secreted [43, 121]. This indicates that the chaperone enhances secretion efficiency but is not absolutely required. Trülzch *et al*. modified Yersinia’s large 67 kb virulence plasmid, which encodes T3SS-associated genes, to only contain the structural components of the T3SS without any known chaperones or effectors [122]. They then expressed effectors with or without chaperones in trans. They showed that *Yersinia enterocolitica* T3SS effectors YopO, YopP, YopM and YopQ were secreted in the absence of any known or orphan chaperones. While this system did not rule out the contribution of genomically encoded chaperones, in the decades since then only YopO has been shown to have a chaperone [45]. Whether as-of-yet unidentified chaperones are required for the other three effectors remains an open question. Ernst *et al*. sought to explore the same scenario in *Shigella* by constructing a strain where all known class I chaperones were deleted [123]. Most *Shigella* effectors were secreted at similar levels between wild-type and chaperone-deletion strains. They proceeded to express effectors with only the core T3SS apparatus in a laboratory strain of *E. coli* and still observed efficient secretion of most effectors. The plate-based screen approach used to detect secreted effectors is vulnerable to false positives, especially in the case of bacterial cell lysis, potentially conflating secreted effectors with proteins only present in the bacterial cell. Thus, it may be prudent to validate these findings by testing effector secretion and function during infection using a chaperone-deletion strain.

Can these findings be explained by other proteins moonlighting as chaperones when a chaperone is absent or deleted, or are these studies enough to fundamentally shift our understanding of the importance of chaperones in effector secretion? Of note, chaperones have not been identified for many T3SS effectors, including over 80% of those from *Salmonella*. While these effectors may require chaperones that have not yet been discovered, it is interesting to note that some effectors can be secreted based on their N-terminal secretion signal alone [124]. Therefore, while chaperones may increase the efficiency of secretion by increasing the stability of the T3SS substrate, improving its affinity for the secretion system itself or regulating the temporal hierarchy of secretion, they may not be strictly necessary.

Perhaps these findings point to a distinct T3SS-independent mechanism of secretion, one that is implicitly chaperone-independent. For example, analyses of outer membrane vesicles (OMVs) of *S. typhimurium* show that SPI-1 T3SS effectors are not only present on OMVs that pinch off bacterial membranes but that these effectors on OMVs can enter host cytoplasm and remain biologically active independent of the T3SS [125, 126]. The same has been shown in enterohaemorrhagic *E. coli* [127]. Perhaps the ability to secrete potent virulence factors in a less spatiotemporally stringent way than injectisome secretion gives the bacteria a selective advantage. However, it is unclear whether these phenomena can be observed in infection conditions.

### Outlook

Although the T3SS was discovered over 30 years ago [128], and despite its central importance for the virulence of clinically and agriculturally relevant pathogens, many questions remain about the roles that chaperones play in the mechanics of secretion. It is clear that for needle and translocon components, chaperones suppress the activity of their clients in the bacterial cell, promote their stability and instil a hierarchy of secretion. New evidence is emerging about the dynamic nature of chaperones at the cytoplasmic base of the T3SS that complicates our understanding of the targeting of clients to the injectisome. The role of class I chaperones in effector secretion is less clear. It is striking that there are significant numbers of orphan effectors without known chaperones. It is intriguing to question whether their chaperones are yet-to-be discovered or whether chaperones are not needed in all situations and may not have co-evolved with certain effectors. If the latter is true, it will be informative to see whether these will prove to be the exceptions to the rule, as shown for *Yersinia* [122], or whether a majority of effectors can be secreted without chaperones, as seems to be the case for *Shigella* [123]. Experiments expressing core T3SS structural components and individual effectors without their chaperones in heterologous systems have been informative. Expanding such studies to other species, together with direct testing of chaperone-deletion strains, may help determine the extent to which effector secretion depends on cognate chaperones. Determining the importance of chaperones will be of particular interest to researchers working on recombinant protein expression. Secretion of heterologous proteins in bacteria via the T3SS may reduce cytotoxicity to the bacteria and simplify purification steps by forgoing the need for bacterial cell lysis [129]. Additionally, a detailed understanding of the requirements for secretion, including the characterization of a simplified system for secretion requiring fewer components, could be important for use in delivering proteins as vaccines.

## Abbreviations

CBD: chaperone-binding domain
EPEC: enteropathogenic *Escherichia coli*
MHCII: major histocompatibility complex class II
OMVs: outer membrane vesicles
PMF: proton motive force
Sct: secretion and cellular translocation
SP: sorting platform
SPI: *Salmonella* pathogenicity island
TPR: tetratricopeptide repeats
T3SS: type III secretion system
